# Corrected and Republished from: “Isolation and Characterization of the First Freshwater Cyanophage Infecting *Pseudanabaena*”

**DOI:** 10.1128/jvi.00405-23

**Published:** 2023-04-19

**Authors:** Dong Zhang, Fang You, Yiliang He, Shu Harn Te, Karina Yew-Hoong Gin

**Affiliations:** a NUS Environmental Research Institute, National University of Singapore, Singapore; b Department of Civil and Environmental Engineering, National University of Singapore, Singapore; c School of Environmental Science and Engineering, Shanghai Jiao Tong University, Shanghai, China; St. Jude Children's Research Hospital

**Keywords:** cyanophages, freshwater, full genome, isolation, *Pseudanabaena*

## Abstract

Cyanobacteria are the major primary producers in both freshwater and marine environments. However, the majority of freshwater cyanophages remain unknown due to the limited number of cyanophage isolates. In this study, we present a novel lytic freshwater cyanophage, PA-SR01, which was isolated from the Singapore Serangoon Reservoir. To our knowledge, this is the first isolate of a cyanophage that has been found to infect the cyanobacterium *Pseudanabaena*. PA-SR01 has a narrow host range, a short latent period, and is chloroform sensitive. PA-SR01 is a member of *Siphoviridae* with a long noncontractile tail. It is a double-stranded DNA virus with a 137,012-bp genome. Functional annotation for the predicted open reading frames (ORFs) of the PA-SR01 genome identified genes with putative functions related to DNA metabolism, structural proteins, lysis, host-derived metabolic genes, and DNA packaging. Out of 166 predicted ORFs, only 17 ORFs have homology with genes with known function. Phylogenetic analysis of the major capsid protein and terminase large subunit further suggests that phage PA-SR01 is evolutionary distinct from known cyanophages. Metagenomics sequence recruitment onto the PA-SR01 genome indicates that PA-SR01 represents a new evolutionary lineage of phage which shares considerable genetic similarities with phage sequences in aquatic environments and could play key ecological roles.

**IMPORTANCE** This study presents the isolation of the very first freshwater cyanophage, PA-SR01, that infects *Pseudanabaena*, and fills an important knowledge gap on freshwater cyanophages as well as cyanophages infecting *Pseudanabaena*.

## INTRODUCTION

Cyanobacteria play important roles in primary production and trophic interactions. They are the dominant autotrophs in most aquatic environments, such as freshwater and marine environments (1). Viruses infecting cyanobacteria are referred to as cyanophages and can play major roles in the dynamics, genetic diversity, and structure of cyanobacterial communities (2–4). Compared to marine cyanophages, which have been widely studied (5), there are very limited studies on freshwater cyanophages (6, 7).

To better understand the biological interactions and evolutionary relationships between cyanophages and their host, cyanophage whole-genome sequences could provide a solid platform to elucidate such relationships (8–12). At the same time, as metagenomics becomes a more prevalent approach to monitoring environmental cyanophage diversity, genomic sequences of cultured cyanophages are needed for more precise annotation of the viral metagenome. Most viral sequences in metagenomic databases cannot be allocated putative functions and there are still many viral contigs of unknown identity in metagenomes (13, 14). This further strengthens the case for the need to acquire more genomic sequences of new cyanophage isolates, especially in the case of freshwater cyanophages.

Based on their morphological differences, cyanophages are generally categorized within three families, the *Myoviridae*, *Podoviridae*, and *Siphoviridae*, which belong to the order *Caudovirales. Myoviridae* have long contractile tails, *Podoviridae* have short noncontratile tails, while *Siphoviridae* have long flexible tails (15). Only one tailless cyanophage has been isolated to date (16).

PA-SR01 infects and lyses freshwater *Pseudanabaena* strain KCZY-C8. Despite reports of several cases of *Pseudanabaena* presence in cyanobacterial blooms (17–19), there have been no *Pseudanabaena*-infecting phages isolated to date to the best of our knowledge. PA-SR01 is the first cyanophage infecting and lysing *Pseudanabaena*. To explore the biological properties and ecological roles of PA-SR01, we first studied the morphology and infection process, followed by sequencing the PA-SR01 genome and performing functional gene annotation. To further understand its environmental presence, recruitment of metagenomics reads onto the PA-SR01 genome was performed and revealed its prevalence in aquatic systems around the globe.

## RESULTS AND DISCUSSION

### Physical properties of phage PA-SR01.

**FIG 1:**
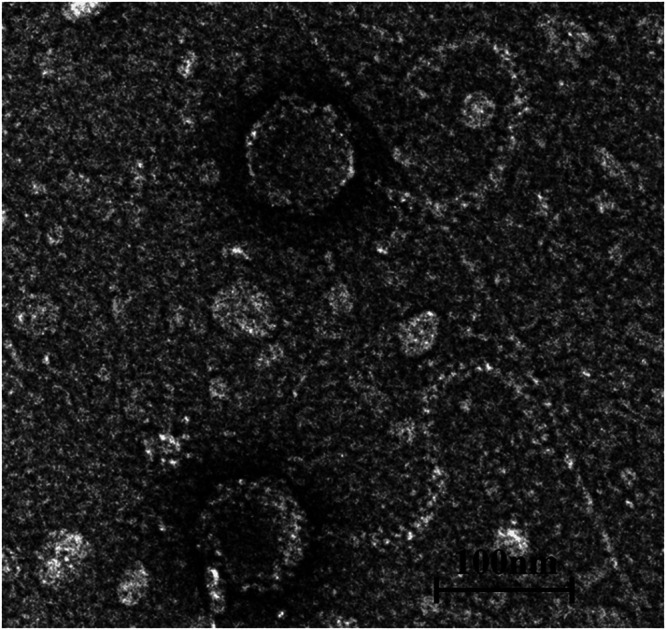
Transmission electron micrograph showing morphological features of PA-SR01.

The transmission electron microscopy images of PA-SR01 phage (Fig. 1) showed numerous virus particles with similar size and morphology. The cross sections of the viral particle appeared hexagonal with a long non-contractile tail attached, indicating that the virus had an icosahedral symmetry and is a member of *Siphoviridae*. The average diameter of viral particles ranged from 88 to 95 nm (mean ± SD = 91 ± 3 nm), with a tail of ~300 nm.

### Host specificity.

PA-SR01 lysed only *Pseudanabaena* strain KCZY-C8 and not the additional 2 *Pseudanabaena* strains and 17 cyanobacterial species (Table 1). Based on this result, we concluded that PA-SR01 is strain specific rather than species specific, and has a narrow host range. To understand whether PA-SR01 infectivity is correlated with geographical location, more *Pseudanabaena* strains will need to be isolated from other tropical water bodies.

**TABLE 1:** List of cyanobacteria used for host range test

Genus	Strain	Origin	Susceptibility
*Cylindrospermopsis*	CS505	Lakes in tropical Queensland	−
*Cylindrospermopsis*	CS511	Lakes in tropical Queensland	−
*Cylindrospermopsis*	Cyl UPR	Upper Peirce Reservoir	−
*Cylindrospermopsis*	CS509	Lakes in tropical Queensland	−
*Cylindrospermopsis*	Cy2.2	Serangoon Reservoir	−
*Cylindrospermopsis*	Cy3.4	Serangoon Reservoir	−
*Limnothrix*	MRS2	Marina Reservoir	−
*Microcystis*	I21	Serangoon Reservoir	−
*Microcystis*	B	Kranji Reservoir	−
*Microcystis*	I1	Serangoon Reservoir	−
*Microcystis*	I31	Serangoon Reservoir	−
*Microcystis*	M1	Marina Reservoir	−
*Microcystis*	K18	Kranji Reservoir	−
*Pseudanabaena*	KCZY-C8	Serangoon Reservoir	+
*Pseudanabaena*	MRS2	Marina Reservoir	−
*Pseudanabaena*	M13A	Marina Reservoir	−
*Synechococcus*	IA	Serangoon Reservoir	−
*Synechococcus*	Cip1	Serangoon Reservoir	−
*Synechococcus*	Cip6	Serangoon Reservoir	−
*Synechococcus*	R4S1	Serangoon Reservoir	−
*Synechococcus*	Cip21	Serangoon Reservoir	−

### Chloroform sensitivity.

**FIG 2:**
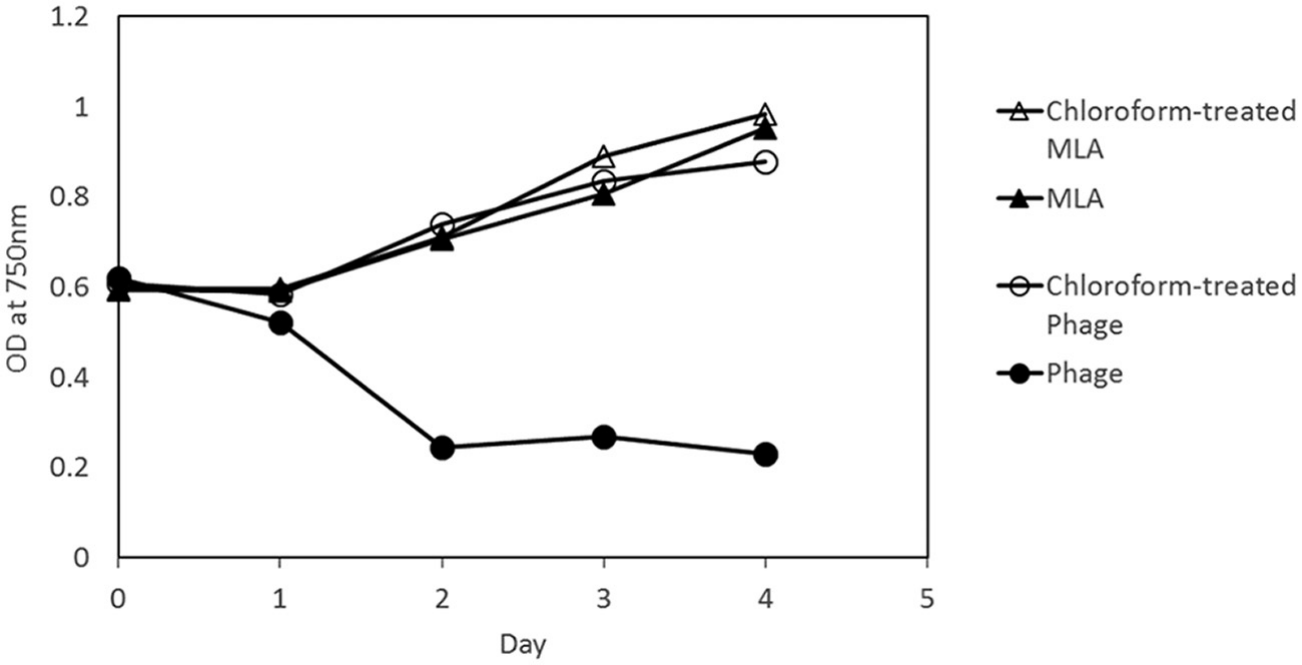
Effect of chloroform on the infectivity of PA-SR01. OD at 750 nm is shown for *Pseudanabaena* cultures grown in untreated (open triangle) or chloroform-treated MLA medium (black triangle), or else inoculated with chloroform-treated (black circles) or untreated viruses (open circles).

The infectivity of PA-SR01 is chloroform sensitive (Fig. 2). Chloroform treatment makes phage lose its infectivity and there is no observable effect on host cell growth with or without addition of chloroform-treated MLA medium. Chloroform sensitivity serves as a first indication of a viral lipid component (20). Chloroform can dissolve lipids that may be structural components of infection mechanisms in lipid-containing phage (21). However, chloroform sensitivity alone does not prove the presence of lipid in viral particles. Further studies are needed to confirm the presence of lipids in PA-SR01. This figure also shows that the latent period of PA-SR01 is approximately 1 day, which is relatively short compared to other freshwater cyanophages such as S-LBS1 (latent period of 4 days), PaV-LD (latent period of 2 days), and S-EIV1 (latent period of 2 days) (9, 10, 12).

### Genomic overview.

**FIG 3:**
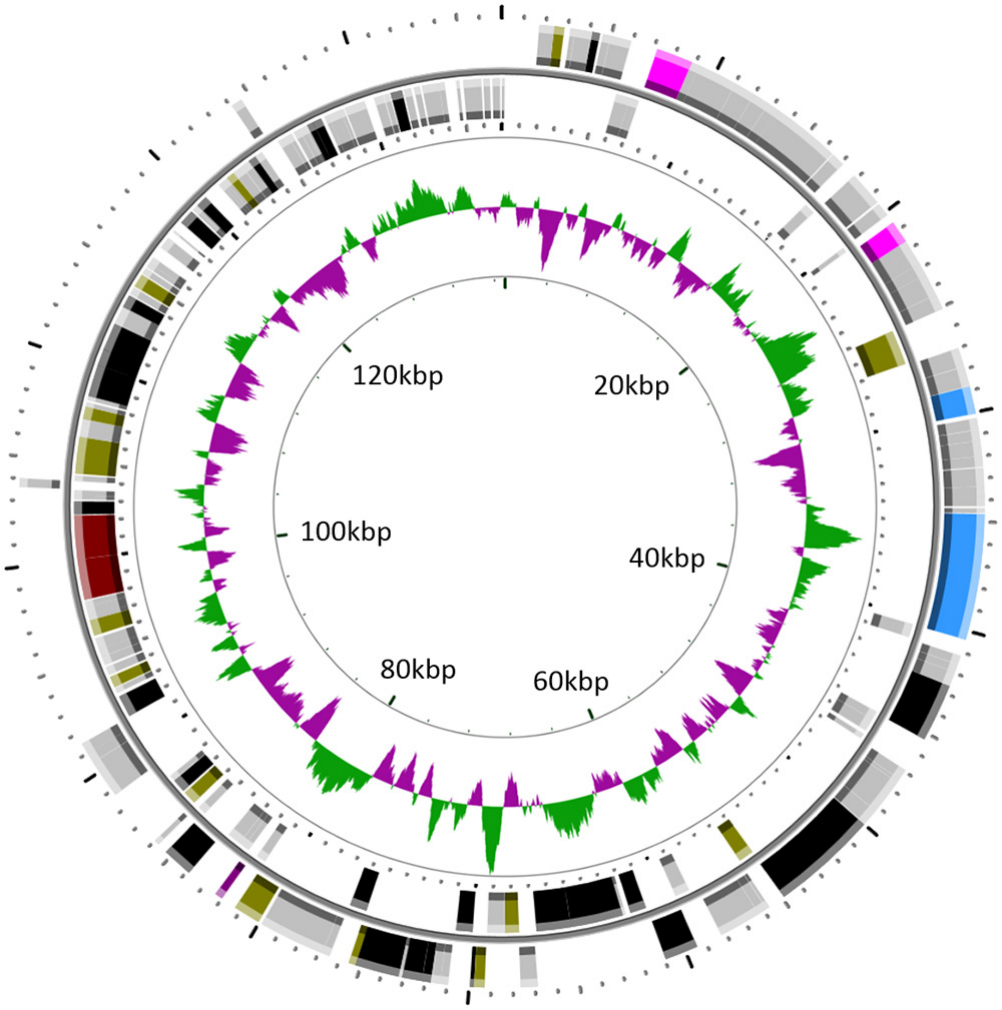
Genomic map of PA-SR01. Circles from outermost to innermost correspond to (i) predicted ORFs (BLASTp, nr database, E value of <0.00001) on forward strand and (ii) reverse strand; (iii) GC content plotted with green representing G+C content and purple representing A+T content. Only ORFs of >100 bp are shown and are colored as follows: black, hypothetical protein; gray, no homolog; pink, DNA packaging; blue, structural protein; crimson, host-derived metabolic gene; brown, DNA metabolism; purple, lysis.

The 137,012-bp genome of PA-SR01 is circularly permutated (Fig. 3) with a GC content of 39.5%. One hundred sixty-six ORFs were predicted, though most ORFs in PA-SR01 did not have homologous genes of known function. In total, 47 ORFs had significant similarity to other sequences, and more than 70% of the ORFs could not be annotated to any homologs. Only 11 ORFs were similar to phage sequences and only 17 were similar to genes of known function (BLASTp; E value cutoff = 10^−5^). Three clustered tRNA genes, tRNA^Met^, tRNA^Asp^, and tRNA^Gly^, were identified (Table 2).

**TABLE 2:** tRNAs predicted with tRNAscan-SE2

tRNA number	tRNA start	tRNA end	tRNA type	Anticodon
1	58579	58502	Met	CAT
2	58497	58427	Asp	ATC
3	58356	58284	Gly	TCC

Genome annotation of PA-SR01 ORFs identified putative genes with functions associated with structural proteins, DNA metabolism, DNA packaging, and lysis (Table 3). HHpred was used to ascribe function to additional ORFs (Table 4). This resulted in the identification of genes encoding putative functions associated with DNA-binding domains (ORF3), Mu-like prophage I protein (ORF24), major capsid protein (ORF32), and PD-(D/E)XK endonuclease (ORF139). In total, 21 ORFs showing homology to genes of known function were obtained.

**TABLE 3:** Predicted ORFs of cyanophage PA-SR01 with similarity to genes of known function

ORF	GenBank ID	Strand	% identity	E value	Putative protein encoded	Organism
12	WP_097778008.1	+	29	1.00E−55	Phage terminase large subunit	Faecalibacterium prausnitzii
29	RTL07602.1	−	38	8.40E−72	DEAD/DEAH box helicase	Candidatus Dependentiae bacterium
41	ADP97718.1	+	21	3.40E−26	Phage tail tape measure protein, family	Marinobacter adhaerens HP15
55	QBQ77753.1	−	35	8.80E−26	Putative group I intron endonuclease	*Escherichia* phage vB_EcoM_WFbE185
66	YP_009100781.1	−	35	3.70E−23	Homing endonuclease	*Shigella* phage Shf125875
68	KKP95496.1	+	30	1.00E−11	Crossover junction endodeoxyribonuclease RuvC	Candidate division TM6 bacterium
77	WP_138275383.1	+	33	6.00E−09	Crossover junction endodeoxyribonuclease RuvC	Candidatus Rhodoluna limnophila
81	WP_050045131.1	+	90	2.00E−218	IS200/IS605 family element transposase accessory protein TnpB	Tolypothrix bouteillei
83	WP_070058383.1	+	51	7.00E−16	Septal ring lytic transglycosylase RlpA family protein	*Marinobacter* sp. X15-166B
90	WP_131120822.1	−	48	7.10E−19	KilA-N domain-containing protein	Westiellopsis prolifica
98	OBQ26706.1	−	39	1.10E−20	Appr-1-p processing protein	Aphanizomenon flos-aquae LD13
103	QBQ73194.1	−	68	5.70E−92	FAD-dependent thymidylate synthase	*Nodularia* phage vB_NspS-kac65v151
107	BBI90448.1	−	34	6.00E−112	Ribonucleotide-diphosphate reductase subunit beta	*Tenacibaculum* phage PTm1
108	WP_018298810.1	−	49	3.20E−198	Ribonucleoside-diphosphate reductase subunit alpha	Fangia hongkongensis
114	PSB68310.1	−	35	6.00E−99	DNA-directed DNA polymerase	Filamentous cyanobacterium CCP1
116	WP_119501154.1	−	30	3.00E−08	Deoxynucleotide monophosphate kinase	*Alteromonas* sp. RKMC-009
125	YP_009042791.1	−	48	1.50E−21	Recombination protein	*Anabaena* phage A-4L

**TABLE 4:** ORFs with distant homology to PA-SR01 identified using HHpred analysis

ORF	Pfam ID^*a*^	Strand	E value	Putative protein encoded
3	PF10544.9	+	5.2E−16	T5orf172 domain
24	PF10123.9	+	4.5E−18	Mu-like prophage I protein
32	PF03864.15	+	1.5E−30	Phage major capsid protein E
139	PF11645.8	−	0.0000018	PD-(D/E)XK endonuclease

a Pfam ID data can be found at https://pfam.xfam.org.

Seven rho-independent terminators were predicted by Findterm (Table 5); 6 are downstream of ORFs with unknown function (ORF30, ORF33, ORF61, ORF62, ORF73, and ORF102) and one is downstream of a gene predicted to encode a Kila-N domain-containing protein (ORF90).

**TABLE 5:** PA-SR01 rho-independent terminators predicted by Findterm

Terminator	Start	End	Length (bp)	Strand	Energy (kCal)	Upstream ORF	Distance to ORF
Term1	26973	26924	50	+	−18.8	30	21
Term2	30131	30088	44	+	−17.3	33	139
Term3	61015	60972	44	+	−21.5	61	2
Term4	61988	61943	46	+	−22.7	62	104
Term5	71819	71770	50	+	−20.9	73	89
Term6	86765	86805	41	−	−16.9	90	67
Term7	95947	95996	50	−	−18	102	87

Despite having the morphological features of *Siphoviridae*, the genomic content of PA-SR01 did not share much similarity with known *Caudovirales*. Out of 166 ORFs predicted, merely 6 ORFs were homologous to genes from known *Caudovirales*.

### Structural genes.

**FIG 4:**
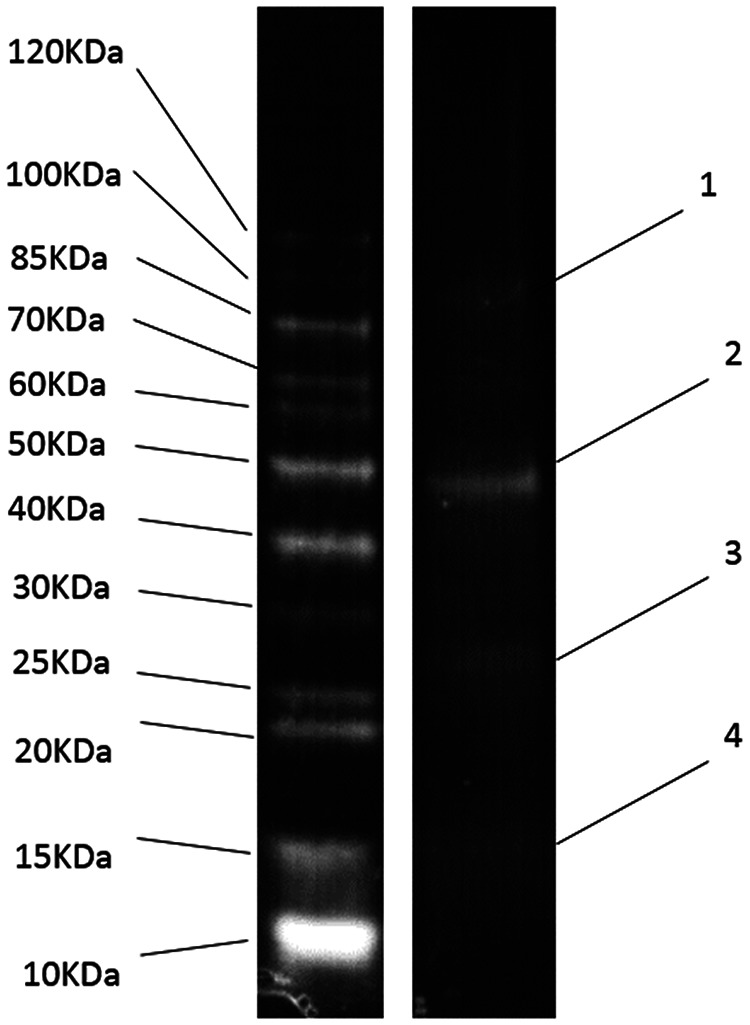
SDS-PAGE for structural proteins of PA-SR01.

ORF41 is the only ORF in PA-SR01 encoding tail tape measure protein (TMP), which is a tail-associated protein. Tail tape measure protein of tailed phages determines the tail length and enables DNA transition into the host cell during infection (22). Despite the name suggesting its widespread presence in tail phage genomes, the tail tape measure protein-encoding gene has, nevertheless, also been observed to be present in tailless phages (10). This indicates that tail tape measuring protein is not unique to tailed phages. Very few sequences encoding known phage structural proteins were found in the PA-SR01 genome other than the major capsid protein (ORF32). This further supports the structural distinction of PA-SR01 from other known phages. With SDS-PAGE analysis, 4 structural proteins of about 16, 28, 47, and 99 kDa (Fig. 4) were resolved. ORF32, encoding the major capsid protein, can be matched to the 47-kDa band. ORF46, ORF9, and ORF101, encoding hypothetical proteins, can be matched to the 99-kDa, 28-kDa, and 16-kDa bands, respectively. However, 4 structural proteins do not represent the full picture, as 13 structural proteins have been identified in phage with a similar genomic size (10, 23). To obtain a more thorough understanding of structural proteins in PA-SR01, a mass spectrometer approach is needed.

### Host-derived genes.

Host-derived metabolic genes are commonly present in cyanophages and play important roles in interactions between cyanophage and their host (24). For example, a survey of 33 cyanophages revealed that psbA was found in 88% of the cyanophage genomes and 50% of the cyanophages contained both psbA and psbD genes (25). Besides photosynthetic genes, other host-derived genes have also been found that are responsible for phycobilisome degradation, carbon metabolism, phosphate uptake, and nucleotide biosynthesis (11, 26–28). The only host-derived metabolic gene identified in PA-SR01 genome is ribonucleotide-diphosphate reductase (RNR) (24). This suggests that PA-SR01 is evolutionary distinct from known cyanophages and could have its own special metabolic genes that require further study.

In PA-SR01, ORF107 and ORF108 were found homologous to ribonucleotide-diphosphate reductase (RNR) subunit alpha and beta, respectively. The RNR gene product can reduce ribonucleotide diphosphate to deoxy-ribonucleotide diphosphate, which is a precursor of DNA (26). Cyanophage can thus make use of RNRs to degrade host DNA to provide building blocks for synthesizing genomes of phage progeny. RNR genes are considered essential for the rapid replication found in lytic phage (29), and this could be a contributing factor to the short latent period of PA-SR01.

### Nucleotide metabolism.

Besides RNR, there were several genes identified that are involved in nucleotide metabolism. The PA-SR01 genome encodes a homolog (ORF103) of FAD-dependent thymidylate synthase (ThyX) that produces thymidylate (dTMP) *de novo* from dUMP (30). The importance of ThyX in phage genome replication has been demonstrated in double-stranded DNA virus (31). In the PA-SR01 genome, another gene possibly involved in nucleotide metabolism is ORF116, encoding a homolog of deoxy-nucleotide monophosphate kinase which may phosphorylate dGMP, dTMP, and 5-hydroxymethyl-dCMP to be used in producing new viral DNA genomes (32). Both ThyX and deoxy-nucleotide monophosphate kinase might contribute to phage genome replication in PA-SR01. dTMP produced by ThyX could be phosphorylated to dTDP, which could be further phosphorylated by nucleoside-diphosphate kinase (NDPK) to form dTTP, a monomer that can be utilized by DNA polymerase (ORF114) to generate long-chain DNA molecules. However, no homologs of NDPK were found in the PA-SR01 genome, and thus further studies are needed to better understand the detailed nucleotide biosynthesis strategy of PA-SR01.

### Insertion element.

PA-SR01 has one ORF showing extremely high similarity to the ORF from cyanobacterium Tolypothrix bouteillei. ORF81 has 90% amino acid sequence similarity to IS200/IS605 family element transposase accessory protein. Such a high sequence similarity is rare in phage genome and suggests that this ORF originated from recent horizontal gene transfer. Similar insertion sequences (IS) have been observed in other phage genomes (11, 33) and their functions remain unknown. IS elements are rare in phage genomes and are considered disadvantageous for bacteriophage propagation as they could disrupt the efficiency of phage genome organization (33). This also supports the hypothesis that ORF81 came from recent horizontal gene transfer, as it is less likely for a phage genome with IS elements to propagate and pass on its gene over many generations compared to phage without IS elements.

### Lysis-associated genes.

The lysozyme homolog is commonly found in cyanophage and is believed to be the functional gene for cell lysis (9, 11, 12). However, no homologs of lysozyme can be found in the PA-SR01 genome; instead, ORF83 encodes a putative septal ring lytic transglycosylase RlpA family protein. Lytic transglycosylases represent a major class of enzymes capable of lysing bacterial cell walls with the same substrate specificity as lysozyme. Across different families of lytic transglycosylases, family 4 has been shown to be involved with bacteriophage-induced lysis (34). Rare lipoprotein A (RlpA) was found to be a new lytic transglycosylase with strong preference for naked glycan strands (35). ORF83, encoding a homolog of the RlpA family, could be the key gene responsible for cell lysis. This suggests that PA-SR01 adopts a different lysis strategy from known cyanophages and that PA-SR01 is likely to be evolutionary distinct.

### PA-SR01, a new evolutionary lineage of cyanophage.

PA-SR01 represents a new evolutionary lineage of cyanophage based on its genomic content. There is a lack of structural gene similarity between the PA-SR01 genome and other phage genomes, with the exception of the major capsid protein (ORF32) and tail-tape measuring protein (ORF41). Besides structural distinction, PA-SR01 adopts a different lysis strategy from other cyanophages, based on the fact that lytic transglycosylase instead of lysozyme is found in the PA-SR01 genome.

**FIG 5:**
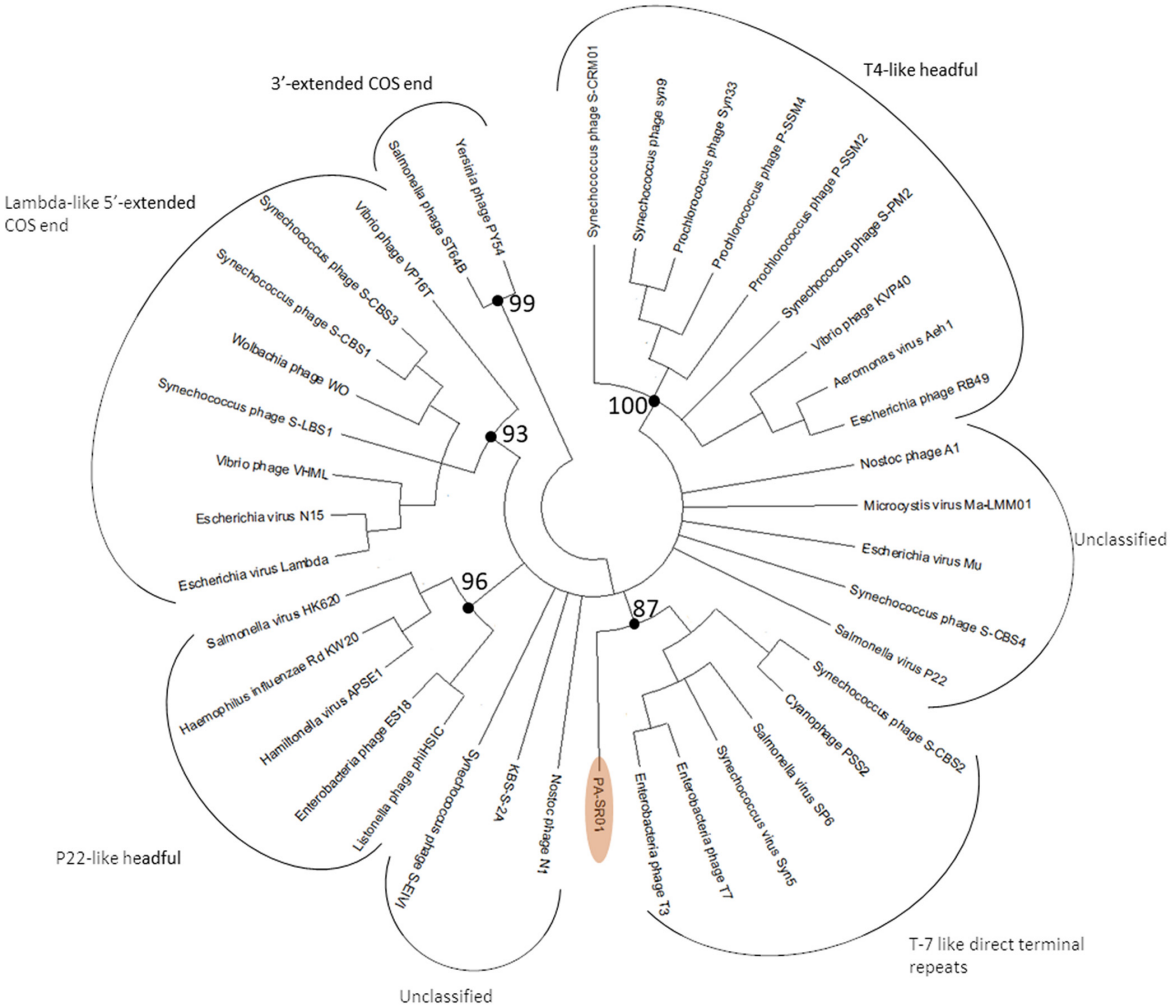
Maximum likelihood amino acid tree of the viral terminase large subunit (terL). Bootstrap values are indicated (100 replicates).

Phylogenetic analysis of the terminase large subunit (terL) and major capsid protein shows that PA-SR01 is evolutionary distinct from other cyanophage isolates. Although PA-SR01 terL is related to T7-like phages, it does not fall within the group of T7-like phages (Fig. 5). Furthermore, the amino acid sequence percentage identity shared between PA-SR01 terL and S-CBS2 terL is merely 26%. The BLASTP result of PA-SR01 terL showed much greater similarity to noncyanophage terL sequences, indicating an evolutionary divergence of terL in PA-SR01.

**FIG 6:**
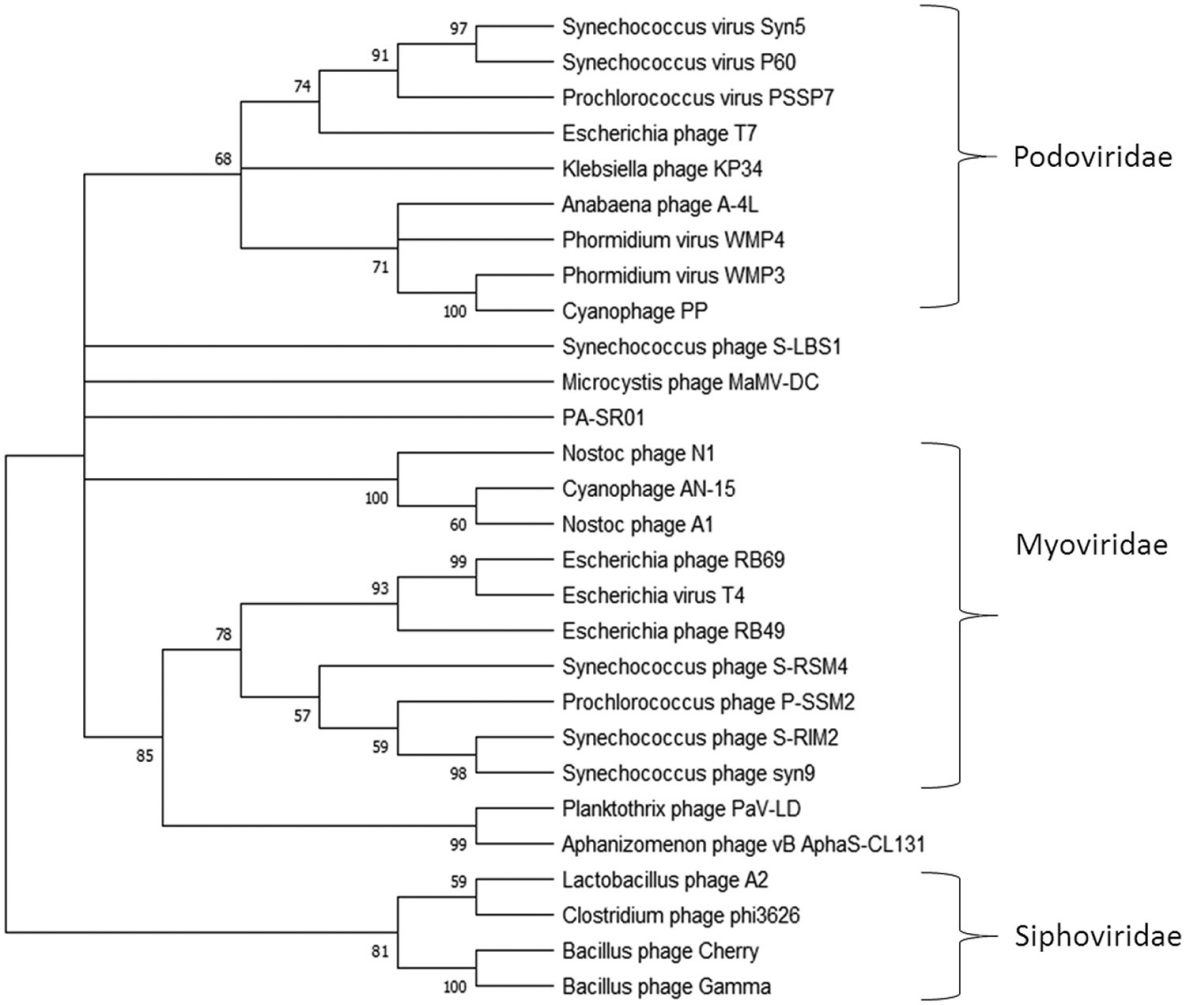
Maximum likelihood amino acid tree of the viral major capsid protein. Bootstrap values are indicated (100 replicates).

Maximum likelihood amino acid tree of the major capsid protein provides further evidence that PA-SR01 is evolutionarily distinct from other cyanophage isolates (Fig. 6). A majority of the phages fall within the three main groups, *Myoviridae*, *Siphoviridae*, and *Podoviridae*, respectively. However, PA-SR01 does not fall within any of the clades and represents an independent branch, providing further support of the evolutionary divergence of PA-SR01 from other phages.

### PA-SR01 sequence similarities in the environment.

**FIG 7:**
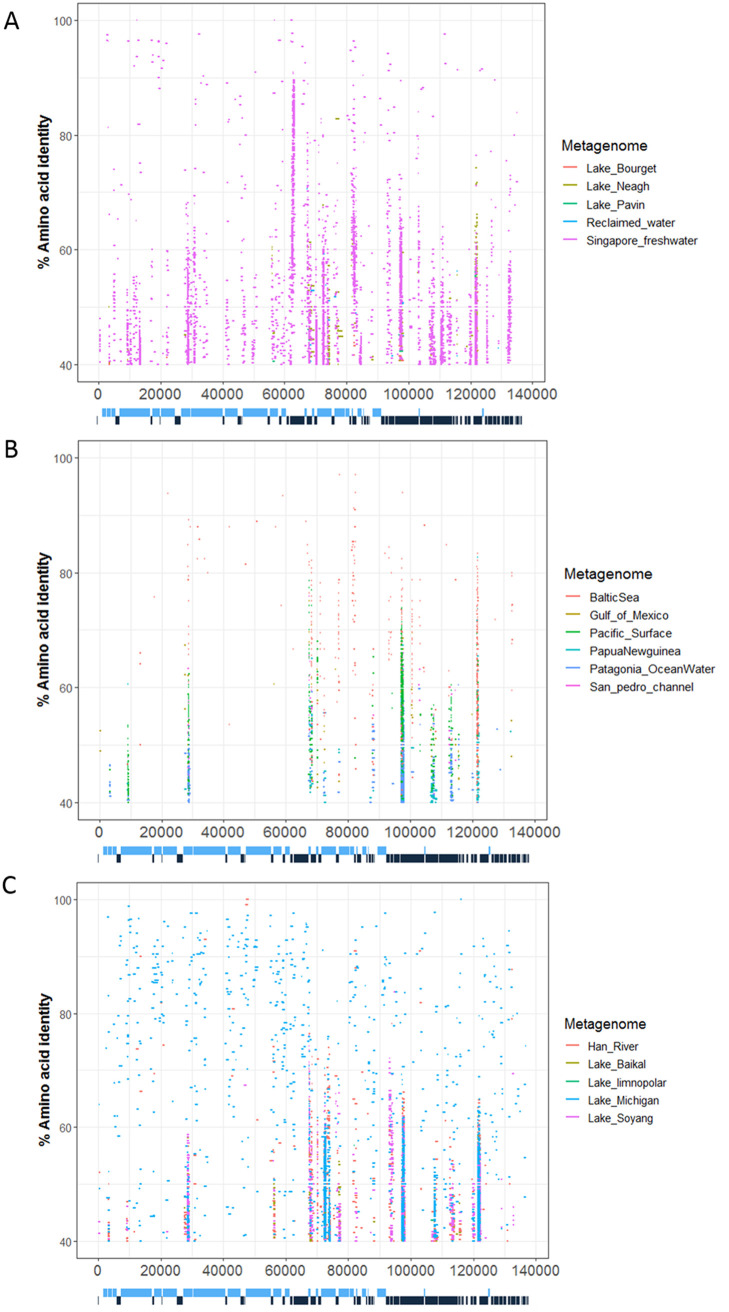
Prevalence of viral sequences similar to PA-SR01 in environmental viral metagenomic data. Fragment recruitment of reads from environmental viral metagenomic data onto the genome of PA-SR01. Each horizontal line represents a read recruited from one of the following publicly available metagenomics data sets: (A) freshwater viral metagenome: Lake Pavin, Lake Bourget, Lake Neagh, reclaimed water virus, and Singapore urban freshwater; (B) marine viral metagenomes: Baltic Sea, Papua New Guinea, Patagonia, Gulf of Mexico, San Pedro Channel, and Pacific Ocean surface; (C) freshwater viral metagenome: Lake Baikal, Lake Limonopolar, Lake Michigan, Lake Soyang, and Han River.

The widespread occurrence of viral sequences similar to PA-SR01 in the environment is shown by the recruitment of metagenomics reads onto the translated PA-SR01 genome. Both marine and freshwater environments were investigated in this analysis (Fig. 7A to C), and 146 ORFs were mapped with at least with one freshwater metagenome and 106 ORFs were mapped with multiple freshwater metagenomes. Twenty-six ORFs were extensively mapped to freshwater metagenomes. Fifty-eight ORFs were mapped to at least one marine metagenome and twenty-two ORFs were mapped across several marine metagenomes. This indicates that PA-SR01-like phages are much more prominent in freshwater. Seven ORFs (ORF12, ORF29, ORF64, ORF103, ORF114, ORF121, and ORF134) were extensively mapped with marine metagenomes and they were all extensively mapped with freshwater metagenomes as well. This suggests that phages adopting similar packaging strategies (e.g., ORF12 encoding a terminase large subunit) and similar DNA metabolism (e.g., ORF29 encoding a DEAD/DEAH box helicase, ORF114 encoding a DNA-directed DNA polymerase, and ORF103 encoding an FAD-dependent thymidylate synthase) are widespread in aquatic environments.

The FAD-dependent thymidylate synthase ThyX (ORF103) and a hypothetical protein (ORF134) have the most recruited sequences across both marine and freshwater metagenomes. ThyX is a key gene in double-stranded phage genome replication, suggesting that phages with similar DNA replication strategy are widespread in aquatic environments and ThyX is an important part of phage DNA replication for both marine and freshwater phages. There are also a large number of recruited reads to IS200/IS605 family element transposase accessory protein TnpB (ORF81), located around 80 kbp. In contrast to the marine environment, 5 out of 10 freshwater metagenomes were recruited onto ORF81. As mentioned previously, TnpB was considered disadvantageous for bacteriophage and is not widely present in known cultured phage (11, 33). The recruited reads from multiple metagenomes onto ORF81 suggests that TnpB might not be detrimental for phage propagation or there could be extensive horizontal gene transfer of TnpB gene from host bacteria to phage.

It is clear in Fig. 7 that we observed many more recruited reads from freshwater viral metagenomes than marine. The majority of ORFs across the genome of PA-SR01 have recruited sequences in the metagenome from urban freshwaters in Singapore. This was expected, since PA-SR01 was isolated from a water body in Singapore and it is likely that viral sequences similar to PA-SR01 are widely distributed locally. Surprisingly, metagenomes from Lake Michigan produced a comparable amount of recruited reads on the PA-SR01 genome. This strongly suggests the widespread presence of viral sequences similar to PA-SR01 around the globe.

**FIG 8:**
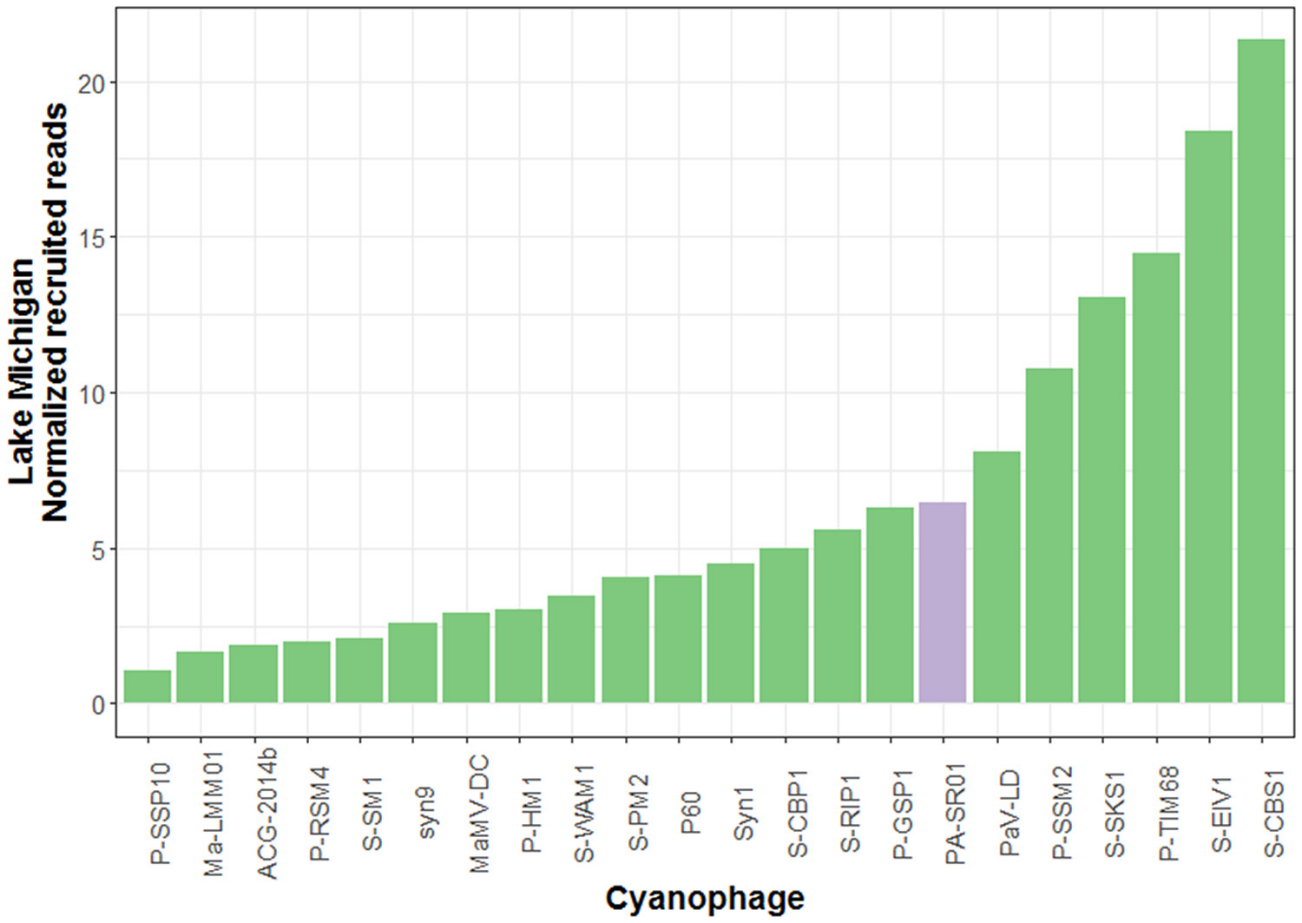
Relative abundance of viral sequences similar to PA-SR01 relative to other cyanophages in Lake Michigan. The number of reads was normalized to the number of genome length of each phage as well as the metagenome database size.

Further evidence for the widespread presence of viral sequences similar to PA-SR01 in the environment is provided by the relative abundance of different cyanophages in the Lake Michigan metagenome (Fig. 8). It is clear that viral sequences similar to PA-SR01 are prevalent in the Lake Michigan metagenome. Although there are several other phages having higher normalized recruited reads than PA-SR01, they are of comparable amount. Furthermore, the number of recruited reads of PA-SR01 is apparently much higher than the majority of other known cyanophages examined in this analysis. Admittedly, the occurrence of PA-SR01 relative to other cyanophages is overestimated due to the fact that only the blast hit with highest E value was recruited. For example, in the list of selected cyanophages there are several P-HM1-like phages, e.g., P-RSM4, P-SSM2, P-TIM68 and Syn1. Significant sequence similarity and core genes are shared among those phages, but only one phage genome would recruit each read, causing the dilution of read numbers assigned to each P-HM1-like cyanophage. Nonetheless, the data indicate that viral sequences similar to PA-SR01 phages are relatively abundant in freshwater environments.

**FIG 9:**
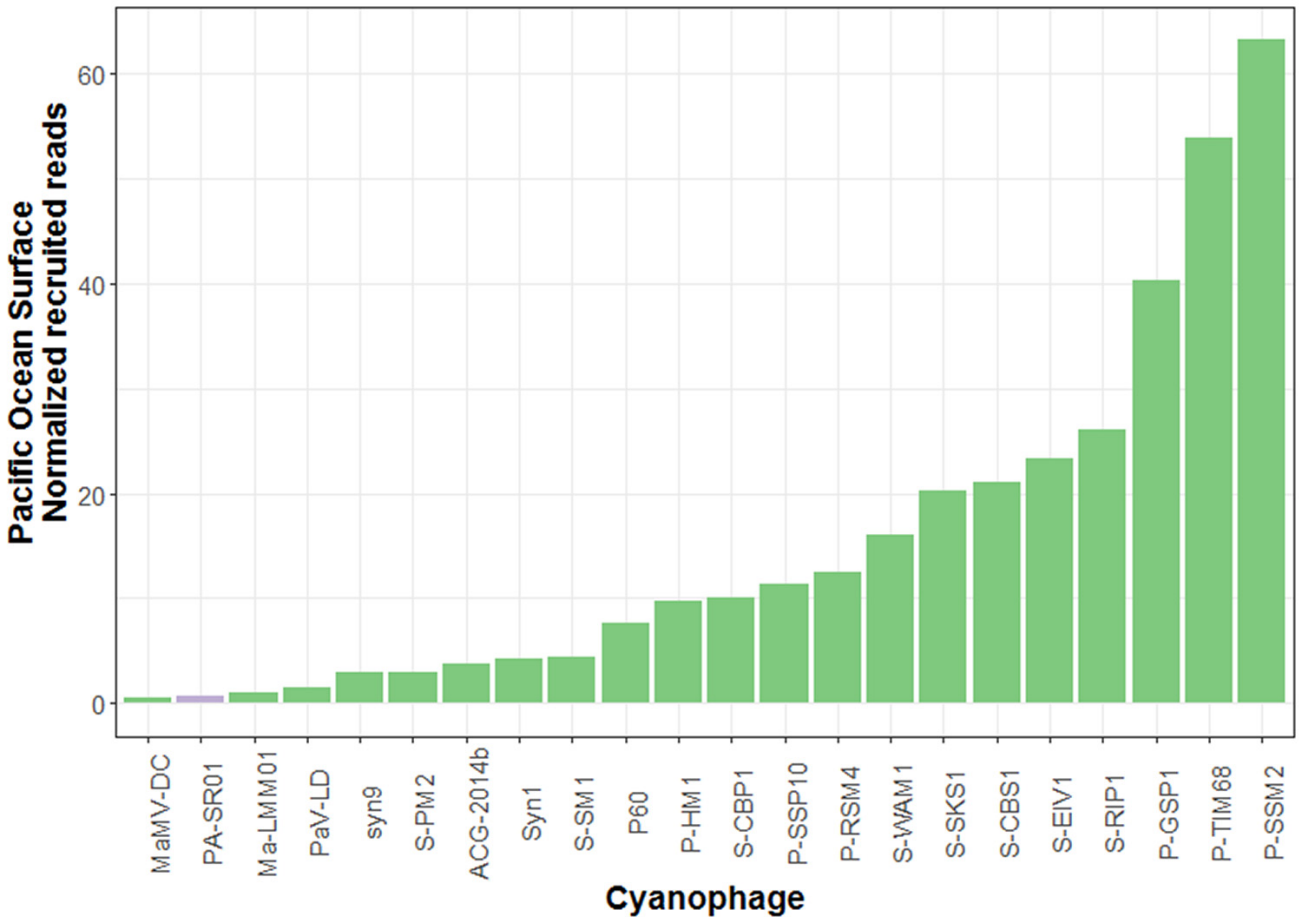
Relative abundance of viral sequences similar to PA-SR01 relative to other cyanophages in the Pacific Ocean surface. The number of reads was normalized to the number of genome length of each phage as well as the metagenome database size.

The relative abundance of viral sequences similar to PA-SR01 in the Pacific Ocean surface water (Fig. 9) is much lower than that in Lake Michigan and is among the least abundant, suggesting that viral sequences similar to PA-SR01 are more prevalent in freshwater environments. Since PA-SR01 was isolated from freshwater, it is more likely to have genes specific to freshwater environments.

In conclusion, this study describes the characteristics and genome of PA-SR01, a novel cyanosiphovirus with a uniquely different set of genes from other known cyanophages. PA-SR01 infects a tropical isolate of *Pseudanabaena* sp. and represents a new evolutionary lineage of cyanophage. Comparative metagenomics data indicate the global prevalence of PA-SR-01-like phages in both freshwater and marine environments. PA-SR01 and related viruses are likely to play major roles in controlling and shaping *Pseudanabaena* populations. Given the large number of genes without homologies in PA-SR01, more work is needed to characterize the phage-host interactions and ecological roles of PA-SR01.

## MATERIALS AND METHODS

### Host cells.

**FIG 10:**
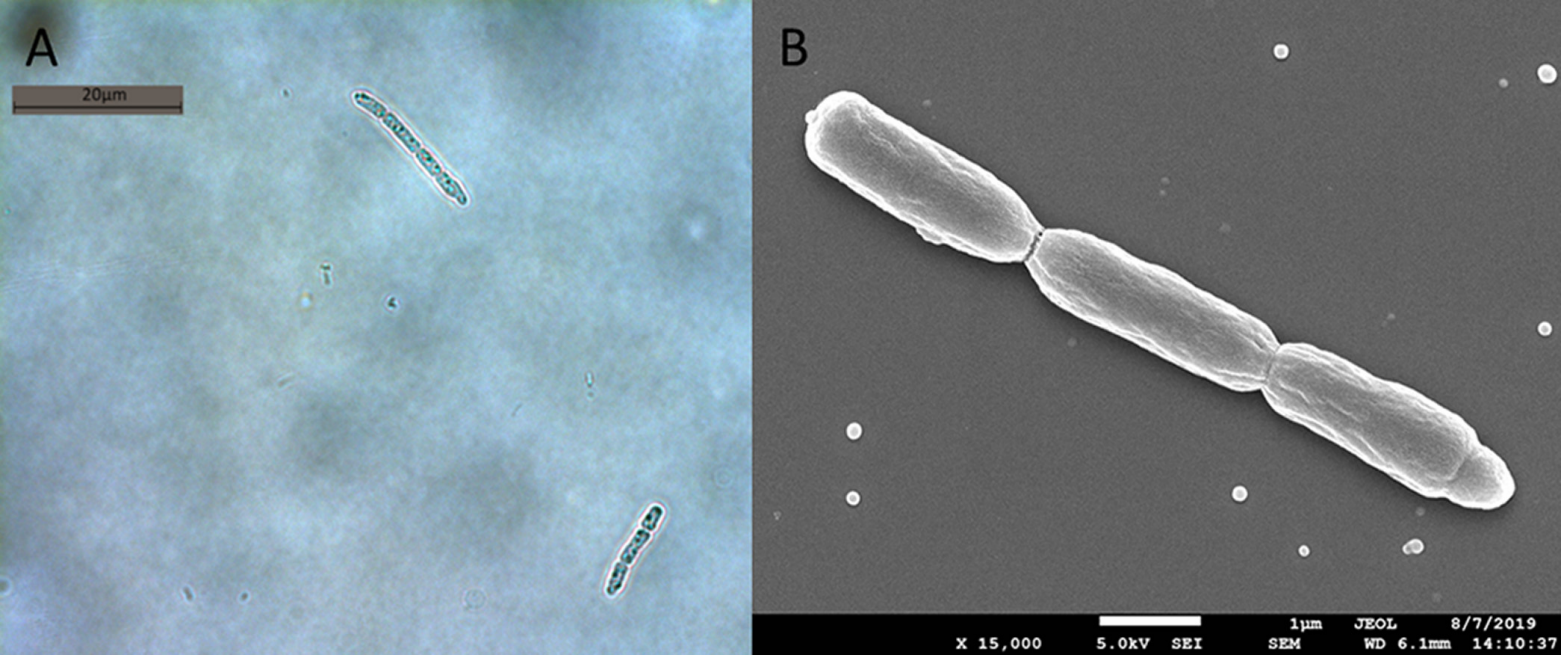
(A) Light microscope image of host strain KCZY-C8. (B) SEM image of host strain KCZY-C8. Both images show that most of the trichomes of KCZY-C8 are in agreement with the morphological characteristics of *Pseudanabaena* reported by Bergey’s Manual of Systematic Archaea and Bacteria (37), as well as other studies (38–40), as follows: cell division in one plane and intercellular breakage of trichome (filament); trichomes are straight; trichomes comprised of 3 barrel-shaped cells (usually 3 to 10 cells per trichome); cell length wider than diameter (diameter = 1μm, length = 3 μm); cell walls are constricted at the junction between adjacent cells.

The *Pseudanabaena* strain KCZY-C8 was isolated in February 2019 from a tropical eutrophic fresh water body (Singapore Serangoon Reservoir) at 1°23′26.2"N 103°54'58.7"E 15 cm below the surface water. The strain was isolated by micropipetting from a surface water sample into sterile MLA medium (36) at 25°C. Identification of the strain was determined to the level of genus following the morphological characteristics (cell shape, dimension, and organization within trichome) reported in Bergey’s Manual of Systematics of Archaea and Bacteria (37) and other studies (38–40). Detailed traits of KCZY-C8 can be found in Fig. 10. We also used partial bacterial 16S rRNA sequence to verify the strain identity (Table 6). The culture was then incubated and maintained in batch culture at 25°C under low radiance (20 μmol photons m^−2^s^−1^) with a 12-h/12-h light/dark cycle.

**TABLE 6:** BLASTN result of host 16S Sequence against NCBI RefSeq database

Strain	Accession no.	% identity	Host 16S sequence
*Pseudanabaena* sp. ABRG5-3	NZ_AP017560.1	93.81	ATCTGNCGTGTCTCAGTCCAGTGTGACTGGTCATCCTCTCAGACCAGTTACCGATCGTCGCCATGGTGTGCCTTTACCACTCCATCTAGCTAATCGGACGCAAGCTCATCTACAGATGATAAATCTTTCACCCGAAGGCATATCCGGTATTAGCAGTCGTTTCCAACTGTTGTCCCGAGTCTGTAGGTAGATTCTTACGCGTTACTCACCCGTAA
*Pseudanabaena* biceps PCC7429	NZ_ALWB01000102.1	91.827	
*Pseudanabaena* sp. UWO310	NZ_SELU01000117.1	91.827	
*Pseudanabaena* sp. Roaring Creek	NZ_LIRE01000034.1	91.827	
*Pseudanabaena* sp. BC1403	NZ_PDDM01000058.1	91.346	
*Pseudanabaena* SR411	NZ_NDHW01000108.1	91.346	

### Cyanophage isolation.

Cyanophage PA-SR01 was isolated from viral concentrates collected from surface water as described above. Briefly, 450 ml of water was filtered through 0.2 μm (Nuclepore) pore size filters. The virus-sized particles in the filtrate were concentrated 100- to 200-fold with a 100-kDa molecular weight (MW) cutoff in ultrafiltration centrifugal tubes (Amicon Ultra-15 centrifugal filter units; Millipore). Viral concentrate was stored at 4°C in dark before any further action. Viral concentrate was serially diluted up to 10^7^ times. PA-SR01 was isolated by adding the aliquots to an exponentially growing culture of *Pseudanabaena* strain KCZY-C8 in a 24-well microtiter plate and incubating at 25°C under low radiance (20 μmol photons m^−2^s^−1^) with 12-h/12-h light/dark cycle for 14 days. Culture lysis was determined by a substantial decrease in optical density at 750 nm (OD_750_) compared with control cultures (41). A clonal viral isolate was obtained by three rounds of extinction dilution (42) in 96-well microtiter plates with exponentially growing *Pseudanabaena* strain KCZY-C8.

### Amplification and purification of PA-SR01.

The cyanophage was amplified by adding 1% (vol/vol) of the virus isolate to 30-ml cultures of *Pseudanabaena* strain KCZY-C8. Both phage-added and control culture were incubated until lysis took place in the culture flasks with phage. The lysates were centrifuged at 15,000 × *g* for 5 min to remove cellular debris. The supernatant containing the majority of viral particles was filtered through a 0.22-μm syringe filter (Minisart syringe filter, Satorius) to remove cellular debris. These purified viral particles were used for subsequent infection experiments.

### Transmission electron microscopy.

Thirty milliliters of PA-SR01 lysate was centrifuged at 15,000 × *g* for 5 min followed by filtering through a 0.22-μm syringe filter to remove the cellular debris. The filtered lysate was centrifuged at 5,000 × *g* with a 100-kDa MW cutoff in ultrafiltration centrifugal tubes (Amicon Ultra-15 centrifugal filter units; Millipore) to increase the phage particle concentration. For staining, 20 μl of gadolinium triacetate (1% wt/wt) was adsorbed to the surface of copper grids at room temperature for 1 min. Excess liquid was blotted from the side of the copper grids with clean filter paper. The grids were viewed and photographed on a JEOL JEM-2100F field emission gun transmission electron microscope at the National University of Singapore Faculty of Chemical and Biomolecular Engineering.

### Host range.

PA-SR01 infectivity was tested against local freshwater isolates of cyanobacteria strains, as well as cyanobacteria obtained from the Commonwealth Scientific and Industrial Research Organisation (CSIRO) culture collection. PA-SR01 phage lysate (1 ml) was added to cultures of exponentially growing cyanobacteria as listed in Table 1. Growth of cyanobacteria cultures without PA-SR01 addition was also monitored to serve as a control. Infectivity was determined by a reduction in OD reading compared to control.

### Chloroform sensitivity.

Chloroform sensitivity of the cyanophage was tested. Filtered lysate (1 ml) was mixed with 1 ml of chloroform followed by shaking manually for 10 min. Chloroform removal was carried out by centrifugation at 4,100 × *g* for 5 min at room temperature. The aqueous phase was transferred to a 1.5 ml microcentrifuge tube and incubated for 6 h at room temperature to remove any remaining chloroform. One milliliter of chloroform was added to treat 1 ml of MLA medium to serve as the control. Chloroform-treated MLA, nontreated MLA, and treated and nontreated virus particles were added to exponentially growing *Pseudanabaena* strain KCZY-C8 cultures and the OD was measured over 6 days.

### DNA extraction, purification, and sequencing.

*Pseudanabaena* strain KCZY-C8 was grown in 300 ml of MLA medium at 25°C under low irradiance (20 μmol photons m^−2^s^−1^) with a 12-h/12-h light/dark cycle until lysis. The lysates were centrifuged at 15,000 × *g* for 5 min to remove cellular debris. The supernatant containing the majority of viral particles was filtered through a 0.22-μm syringe filter (Minisart syringe filter, Satorius) to remove cellular debris. In order to remove free nucleic acid, the lysate was treated with DNase I. The treated lysate was concentrated with a 100-kDa MW cutoff ultrafiltration centrifugal tube (Amicon Ultra-15 centrifugal filter units; Millipore) at 5,000 × *g* to a final volume of 1 ml. QIAamp DNA minikit was used to extract viral DNA with 20 μl of RNase A added in the first step to remove RNA. The cyanophage genome was sequenced using an Illumina High throughput sequencer, with a 150-bp paired-end library constructed using a New England BioLab Next Ultra DNA library prep kit.

### Genome assembly.

The sequencing data were trimmed using BBDuk (version 35.43) to remove adaptors and Phix reads. Reads were *de novo* assembled into contigs by MetaSPAdes genome assembler (3.12.0) (43).

### Genome annotation.

The open reading frames (ORFs) were predicted using GeneMarkS (44) and Prodigal (45); where the prediction differed, the longer of the two was kept. Homology searching was performed with BLASTp against NCBI nonredundant (nr) database (accessed in October 2019). Sequences with E values of <10^−5^ were considered to be homologs. HHpred against protein data bank (PDB) and Pfam database were used to predict more distant homologs (46). The genome was analyzed for tRNA genes with tRNAscan-SE 2.0 (47) and for Rho-independent terminators using Findterm (48), with the energy threshold set to −16 kCal. A genomic map was generated with CGview (49).

### SDS-PAGE analysis for structural protein.

Purified PA-SR01 was diluted in SDS buffer (5:1, vol/vol) and heated at 95°C for 5 min. The sample was then resolved by sodium dodecyl sulfate–polyacrylamide gel electrophoresis (SDS–PAGE) using a Mini-PROTEAN Tetra Cell (Bio-Rad Laboratories). The Mini-PROTEAN TGX stain-free precast gel was run in an SDS running buffer (pH 8.3) at 120 V for 1.5 h using a PageRuler unstained protein ladder (Thermo Fisher) for size calibration.

### Phylogenetic analysis.

The large terminase subunit (terL) and major capsid protein were compared phylogenetically with those from other cyanophages and bacteriophages (Table 7) using Mega-X software (version 10.1.6). ClustalX was used to align the inferred amino acid sequences with default parameters. Based on the multiple sequence alignment, the Jones-Taylor-Thornton (JTT) model was selected and a maximum likelihood tree was constructed with 100 bootstrap replicates.

**TABLE 7:** Accession numbers of genes used in phylogenetic analysis

Accession no.	Gene	Organism
ADZ31560.1	Terminase large subunit	*Planktothrix* phage PaV-LD
AHV82220.1	Terminase large subunit	*Synechococcus* phage S-EIVl
YP_004508468.1	Terminase large subunit	*Synechococcus* phage S-CRM01
YP_717790.1	Terminase large subunit	*Synechococcus* phage syn9
YP_214662.1	Terminase large subunit	*Prochlorococcus* phage P-SSM4
YP_214360.1	Terminase large subunit	*Prochlorococcus* phage P-SSM2
CAF34164.1	Terminase large subunit	*Synechococcus* phage S-PM2
NP_899601.1	Terminase large subunit	*Vibrio* phage KVP40
AAQ17878.1	Terminase large subunit	*Aeromonas* virus Aeh1
NP_891724.1	Terminase large subunit	*Escherichia* phage RB49
AND75577.1	Terminase large subunit	*Nostoc* phage A1
Q9T1W6.1	Terminase large subunit	*Escherichia* virus Mu
YP_063734.1	Terminase large subunit	*Salmonella* virus P22
NP_853601	Terminase large subunit	*Salmonella* virus SP6
P03694	Terminase large subunit	*Enterobacteria* phage T7
P10310	Terminase large subunit	*Enterobacteria* phage T3
YP_224236	Terminase large subunit	*Listonella* phage phiHSIC
YP_224140	Terminase large subunit	*Enterobacteria* phage ES18
NP_050979	Terminase large subunit	*Hamiltonella* virus APSE1
P44184	Terminase large subunit	Haemophilus influenzae phage KW20
NP_112076	Terminase large subunit	*Salmonella* virus HK620
AAA96534	Terminase large subunit	*Escherichia* virus lambda
NP_046897	Terminase large subunit	*Escherichia* virus N15
NP_758915	Terminase large subunit	*Vibrio* phage VHML
BAA89621	Terminase large subunit	*Wolbachia* phage WO
AAQ96470	Terminase large subunit	*Vibrio* phage VP16T
NP_700375	Terminase large subunit	*Salmonella* phage ST64B
NP_892047	Terminase large subunit	*Yersinia* phage PY54
YP_004323723.1	Terminase large subunit	*Prochlorococcus* phage Syn33
BAF36209.1	Terminase large subunit	*Microcystis* virus Ma-LMM01
AND75488.1	Terminase large subunit	*Nostoc* phage N1
ADP06606.1	Terminase large subunit	*Synechococcus* phage S-CBS1
ADF42459.1	Terminase large subunit	*Synechococcus* phage S-CBS3
ADF42432.1	Terminase large subunit	*Synechococcus* phage S-CBS2
AEX55977.1	Terminase large subunit	*Synechococcus* phage S-CBS4
ACT65564.1	Terminase large subunit	Cyanophage PSS2
AGH57666.1	Terminase large subunit	Cyanophage KBS-S-2A
ABP87967.1	Terminase large subunit	*Synechococcus* virus Syn5
ATS93174.1	Terminase large subunit	*Synechococcus* phage S-LBS1
YP_009173820.1	Terminase large subunit	*Synechococcus* virus P60
ABC46474.1	Major capsid protein	Cyanophage AN-15
ABI33181.1	Major capsid protein	*Phormidium* virus WMP4
YP_001285797.1	Major capsid protein	*Phormidium* virus WMP3
YP_009042803.1	Major capsid protein	*Anabaena* phage A-4L
AND75579.1	Major capsid protein	*Nostoc* phage A1
ADZ31580.1	Major capsid protein	*Planktothrix* phage PaV-LD
YP_008766991.1	Major capsid protein	Cyanophage PP
YP_009217771.1	Major capsid protein	*Microcystis* phage MaMV-DC
AND75491.1	Major capsid protein	*Nostoc* phage N1
AOO10060.1	Major capsid protein	*Synechococcus* phage S-RIM2
ATS93177.1	Major capsid protein	*Synechococcus* phage S-LBS1
NP_680487.1	Major capsid protein	*Lactobacillus* phage A2
ATW59308.1	Major capsid protein	*Aphanizomenon* phage vB_AphaS-CL131
YP_214206.1	Major capsid protein	*Prochlorococcus* virus PSSP7
ABP87946.1	Major capsid protein	*Synechococcus* virus Syn5
YP_009173806.1	Major capsid protein	*Synechococcus* virus P60
NP_041998.1	Major capsid protein	*Escherichia* phage T7
ABA46378	Major capsid protein	*Bacillus* phage Cherry
YP_338188.1	Major capsid protein	*Bacillus* phage Gamma
YP_003347636.1	Major capsid protein	*Klebsiella* phage KP34
YP_717802.1	Major capsid protein	*Synechococcus* phage syn9
YP_214367.1	Major capsid protein	*Prochlorococcus* phage P-SSM2
NP_891732.1	Major capsid protein	*Escherichia* phage RB49
NP_861877.1	Major capsid protein	*Escherichia* phage RB69
NP_049787.1	Major capsid protein	*Escherichia* virus T4
YP_003097339.1	Major capsid protein	*Synechococcus* phage S-RSM4

### Recruitment of reads to metagenomics.

The presence of viral sequences similar to PA-SR01 in aquatic environments was investigated by recruiting viral metagenomics data onto the genome of PA-SR01 (50). In total, 88 gigabytes of freshwater metagenome data and 173 gigabytes of marine metagenome data were used (Table 8). Briefly, metagenomic data were first made into a BLAST nucleotide database and queried with the predicted protein sequence of PA-SR01 using tBLASTn (E value of ≤10^−5^), which performed a six-frame translation of the subject nucleotide sequence into protein sequence (51). Metagenomics nucleotide reads with a blast hit to PA-SR01 were then extracted from each metagenome and used as query to blast (BLASTx, E value of ≤10^−5^, max_target_seqs = 1) against a viral protein database containing predicted proteins of PA-SR01 phage and another 2,536 bacteriophage genomes from the NCBI Reference Sequence Database (RefSeq; accessed on Jan 2020). If the best hit was related to PA-SR01 instead of the other phages, it was recruited as viral sequences similar to PA-SR01 and mapped onto the genome of PA-SR01, based on percentage identity of amino acid sequence using ggplot2 (52).

**TABLE 8:** SRA accession numbers of metagenomics data used for metagenome mapping

Source	Database size (Gb)	Accession no.	Marine or freshwater
Pacific Ocean surface	76.10	ERR3256930	Marine
	76.10	ERR3256932	Marine
	76.10	ERR3256934	Marine
	76.10	ERR3256936	Marine
	76.10	ERR3256953	Marine
	76.10	ERR3256955	Marine
	76.10	ERR3256957	Marine
	76.10	ERR3256959	Marine
	76.10	ERR3256964	Marine
	76.10	ERR3256973	Marine
	76.10	ERR3256977	Marine
	76.10	ERR3256975	Marine
Baltic Sea	51.10	SRR7254009	Marine
	51.10	SRR7253988	Marine
	51.10	SRR7253990	Marine
	51.10	SRR7253989	Marine
	51.10	SRR7254008	Marine
	51.10	SRR7254007	Marine
	51.10	SRR7254010	Marine
Singapore urban water	40.00	SRR5995660–SRR5995697	Freshwater
Lake Michigan	23.40	SRR1915829	Freshwater
	23.40	SRR1915851	Freshwater
	23.40	SRR1974489	Freshwater
	23.40	SRR1974496–SRR1974508	Freshwater
	23.40	SRR1974510–SRR1974511	Freshwater
	23.40	SRR1974513	Freshwater
	23.40	SRR1974515	Freshwater
	23.40	SRR1296481	Freshwater
	23.40	SRR1302020	Freshwater
	23.40	SRR1301999	Freshwater
	23.40	SRR1974488	Freshwater
	23.40	SRR1974490	Freshwater
	23.40	SRR1974491	Freshwater
	23.40	SRR1974493–SRR1974495	Freshwater
	23.40	SRR1974509	Freshwater
	23.40	SRR1974512	Freshwater
	23.40	SRR1974514	Freshwater
	23.40	SRR1974516–SRR1974517	Freshwater
	23.40	SRR1302010	Freshwater
San Pedro channel	20.60	SRR10600460	Marine
	20.60	SRR10600461	Marine
Patagonia	15.80	SRR5145173–SRR5145178	Marine
Lake Soyang	13.90	ERS2758845	Freshwater
	13.90	ERS2759121	Freshwater
	13.90	ERS2759122	Freshwater
Gulf of Mexico	9.40	SRR11048275	Marine
Han River	7.50	ERS1546404	Freshwater
	7.50	ERS1546406	Freshwater
Papua New Guinea	5.30	SRR5644412–SRR5644431	Marine
Lake Neagh	1.30	SRR2147000	Freshwater
Lake Baikal	0.87	SRR5936590	Freshwater
Lake Limnopolar	0.39	SRR1658897–SRR1658894	Freshwater
Reclaimed water virus	0.38	SRR014585–SRR014589	Freshwater
Lake Bourget	0.33	ERR019478	Freshwater
Lake Pavin	0.37	ERR019477	Freshwater

The BLAST hits number to PA-SR01 was normalized by dividing by the total number of predicted ORFs and the size of the metagenome (in gigabytes), which provides a normalized measure to compare recruitments across metagenomes of different size. Similar recruitment analysis was also performed for other phage genomes (Table 9).

**TABLE 9:** List of cyanophages selected for metagenomics recruitment analysis

Cyanophage	Accession no.
*Synechococcus* phage ACG-2014b	NC_027130
*Synechococcus* phage S-CAM1	NC_020837
*Synechococcus* phage S-CBP1	NC_025456
*Synechococcus* phage S-CBS1	NC_016164
*Synechococcus* phage S-CRM01	NC_015569
*Synechococcus* phage S-PM2	NC_006820
*Synechococcus* phage S-RIP1	NC_020867
*Synechococcus* phage S-SKS1	NC_020851
*Synechococcus* phage S-SM1	NC_015282
*Synechococcus* phage S-WAM1	NC_031944
*Synechococcus* phage syn9	NC_008296
*Synechococcus* virus P60	NC_003390
*Prochlorococcus* phage MED4-184	NC_020847
*Prochlorococcus* phage P-GSP1	NC_020878
*Prochlorococcus* phage P-HM1	NC_015280
*Prochlorococcus* phage P-RSM4	NC_015283
*Prochlorococcus* phage P-SSM2	NC_006883
*Prochlorococcus* phage P-SSP10	NC_020835
*Prochlorococcus* phage P-SSP3	NC_020874
*Prochlorococcus* phage P-TIM68	NC_028955
*Prochlorococcus* phage Syn1	NC_015288
*Prochlorococcus* virus PSSP7	NC_006882
*Microcystis* phage MaMV-DC	NC_029002
*Microcystis* virus Ma-LMM01	NC_008562
*Planktothrix* phage PaV-LD	NC_016564
*Synechococcus* phage S-EIV1	KJ410740.1

### Data availability.

The whole-genome sequence of the phage is available in GenBank under accession number MT234670.
